# Meetings of Societies

**Published:** 1948

**Authors:** 


### Meetings of Societies

### Bristol Medico-Chirurgical Society

### Programme for 1948-49

The provisional programme for the next session is as follows :

October 13th.—Annual General Meeting. Professor H. J. Drew Smythe's Presidential Address.

November.—"**Early Life**", Professor A. V. Neale.

December.—Clinical Meeting.

January.—Professor Milnes Walker.

February.—Mr. V. B. Green Armytage (London).

March.—"**Chemotherapy**", Dr. J. A. Boycott (Taunton).

April.—"**Coronary Thrombosis**", Dr. G. E. F. Sutton ; "**Renal Plication**", Mr. A. V. Adams.

May.—"**Thymectomy for Myastheina Gravis**", Mr. J. Keynes (London).

### Royal Society of Medicine.

### COMBINED MEETING OF SECTIONS OF LARYNGOLOGY AND OTOLOGY BRISTOL, JULY 2ND AND 3RD, 1948.

ON July 2nd, Dr. A. W. Proetz (St. Louis, U.S.A.), read a paper on the physiology of the nose, with particular emphasis on the importance of preserving ciliary function. Recent work which he described adds further evidence to support views on this subject long held by British laryngologists.

Professor J. M. Yoffey described experiments in the investigation of communications between the meningeal spaces and the nasal mucosa. Dyes introduced into the nose, indian ink, and some virus proteins are absorbed into the lymphatics, but do not enter the cranial cavity : but indian ink can pass in the reverse direction. Professor Yoffey remarked that our predecessors wrote of " purging rheum from the brain " by way of the nose. There may be new support for the accuracy of their views. In the afternoon a clinical meeting was held at the General Hospital : over one hundred members attended in addition to visitors. The main business of Saturday morning was a paper from Dr. F. Schiller (Oxford) on "**Purulent Pachymeningitis**" and the results of treatment by penicillin, combined with surgery. Very large doses of penicillin are required, and the results are improving. The lecturer raised the question whether prophylactic penicillin should become a standard procedure before operation on any of the nasal sinuses.

---

### The South Western Laryngological Association.

CLINICAL meetings have been held at Bristol (October 18th, 1947), Bath (January 3rd, 1948), and Torquay (April 3rd). A large number of cases has been presented at each meeting, attendances have been good and the discussions of great interest and value. Mr. Watson Williams has been re-elected to the chair for 1948–49. The next meeting will be at Newport at 2 p.m. on January 8th, 1949.

---

### The Society of Anaesthetists of the South-western Region.

A MOST successful meeting of the Society was held at Torquay on the 25th and 26th June, 1948. Demonstrations were given of the Etherinton Wilson spinal techniques for high and low anaesthesia, of Curare for endoscopy, and of Myanesin. During the meeting time was allowed for discussions on these techniques. Mr. R. H. Belsey, F.R.C.S., was the guest, and he gave a stimulating and provocative address entitled "**Some Problems of Thoracic Anaesthesia as seen by the Surgeon**". This was followed by open discussion and Mr. Belsey replied to the many questions. The next meeting of the Society will be held at Bristol on the 29th and 30th October, 1948. Dr. John Gillies, President of the Association of Anaesthetists of Great Britain and Ireland, will be the guest. Anaesthetists from other regions will be welcome and details may be obtained from the Hon. Secretary, Dr. G. L. Feneley, Avon Lodge, 8 Parry's Lane, Bristol 9.

---

### British Medical Association, Bath Division.

On 16th July, 1948, at the Royal United Hospital, Bath, Professor Astwood of Boston, U.S.A., delivered a lecture on thiouracil in the treatment of thyrotoxicosis. After a survey of his early experimental work he described the results obtained with thiouracil itself, and also with various related preparations and suggested that methyl thiouracil was one of the most satisfactory which he and his assistants had used.

He stressed the lengthy period during which treatment should be maintained, pointing out that to get the best results treatment must be pushed to the length of securing a mild degree of myxoedema. This stage he thought was better assessed by the clinical picture than by estimations of basal metabolic rate.

Professor Astwood indicated that the dangers of leucopenia and agranulocytosis were less serious than had been originally thought. He quoted figures from the Lahey Clinic (in which treatment was almost exclusively surgical), in support of his view that the results of thiouracil treatment compared favourably with those in surgical clinics of the highest standing.

### TORQUAY AND DISTRICT MEDICAL SOCIETY

CLINICAL meetings have been arranged for November 25th, January 13th, February 24th and April 28th. Other fixtures are:

Nov. 11th. Prof. C. Moir: "**Common Obstetrical Difficulties.**"
Dec. 9th. Dr. Kersley: "**Arthritis.**"
Jan. 27th. Mr. M. F. Nicholls: "**Retention of Urine.**"
Feb. 10th. Prof. A. Moncrieff: "**Diseases of the New-born.**"
March 24th. Mr. Belsey: "**Diseases of the Oesophagus.**"
April 14th. Dr. Brooks: "**Chest Disorders.**"

### CARDIFF MEDICAL SOCIETY

Programme:
Nov. 11th. Dr. Mary Crosse: "**The Premature Baby.**"
Dec. 9th. Mr. Dickson Wright.
March 9th. Sir Harold Gillies. (Annual Dinner.)
May meeting. Dr. Geoffrey Hodgson: "**Dermatology.**"
January, February and April meetings to be arranged later.

### WESSEX RAHERE CLUB

THIS club held its first annual dinner at the Royal Hotel, Bristol on October 17th, 1948. Dr. E. R. Cullinan was Guest of Honour and some thirty-six members were present. Membership is open to all Bart's men resident in Somerset, Wiltshire and Gloucestershire. Mr. C. E. Kindersley (Bath) was elected Chairman and Mr. A. Daunt Batemen (3, Circus, Bath) Hon. Secretary for the coming year. Dr. Cullinan gave an amusing discourse on "Present-day Bart's from within". It is hoped that any Bart's men who are interested, and have not yet been in touch with the club, will notify their whereabouts to the Hon. Secretary so that they can be kept informed of future meetings.

